# Long-term outcome and cancer incidence after abdominal aortic aneurysm repair

**DOI:** 10.1007/s00423-022-02670-x

**Published:** 2022-09-12

**Authors:** A. Ettengruber, J. Epple, Th. Schmitz-Rixen, D. Böckler, R. T. Grundmann, DIGG gGmbH

**Affiliations:** 1 Department of Vascular and Endovascular, Surgery University Hospital, Frankfurt am Main, Germany; 2 German Institute for Vascular Health Research (DIGG) of the German Society of Vascular Surgery and Vascular Medicine, Berlin, Germany; 3 Department of Vascular and Endovascular Surgery, University Hospital, Heidelberg, Germany; 4 Department of Vascular Medicine, University Heart Center (UHC), University Hospital Hamburg-Eppendorf, Hamburg, Germany

**Keywords:** Abdominal aortic aneurysm, Endovascular repair, Open repair, Cancer incidence, Long-term survival

## Abstract

**Purpose:**

The influence of cancer development on long-term outcome after elective endovascular (EVAR) vs. open repair (OAR) of non-ruptured abdominal aortic aneurysms (AAA) was investigated.

**Methods:**

Patient survival and cancer incidence were recorded for 18,802 patients registered with the AOK health insurance company in Germany who underwent EVAR (*n* = 14,218) and OAR (*n* = 4584) in the years 2010 to 2016 (men *n* = 16,086, women *n* = 2716). All patients were preoperatively and in their history cancer-free.

**Results:**

30.1% of EVAR and 27.6% of OAR patients (*p* ≤ .001) developed cancer after a follow-up period of up to 9 years (Kaplan–Meier estimated). Patients with cancer had a significantly less favorable outcome compared to patients with no cancer (HR 1.68; 95% CI 1.59–1.78, *p* < .001). After 9 years, the estimated survival of patients with and without cancer was 27.0% and 55.4%, respectively (*p* < .001). Survival of men and women did not differ significantly (HR 0.94; 95% CI 0.88–1.00, *p* = .061). In the Cox regression analysis (adjusted outcomes by operative approach, gender, age, and comorbidities), the postoperative cancer incidence was not significantly different between EVAR and OAR (HR 1.09; 95% CI 1.00–1.18, *p* = .051). However, EVAR showed an increased risk of postoperative development of abdominal cancer (HR 1.20; 95% CI 1.07–1.35, *p* = .002). 48.0% of all EVAR patients and 53.4% of all OAR patients survived in the follow-up period of up to 9 years. This difference was not significant (HR 0.96; 95% CI 0.91–1.02, *p* = .219).

**Conclusion:**

Cancer significantly worsened the long-term outcome after EVAR and OAR, without significant differences between the two repair methods in the overall cancer incidence. However, the higher abdominal cancer incidence with EVAR can affect quality of life including oncological therapy and therefore should be considered when determining the indication for surgery, and the patient should be informed about it.

In the randomized EVAR trial 1, Patel et al. [1] saw an increased cancer mortality in the EVAR group compared with open repair. They suggested that a possible increase in cancer deaths in the EVAR group in very late follow-up (> 8 years) merits further consideration. In a large population-based cohort study, Markar et al. [2] confirmed this observation. They saw an increased risk of abdominal and all cancer after EVAR compared with OAR, but this was not apparent for lung carcinoma. Markar et al. [2] suggested that radiation exposure during fluoroscopy may be associated with an increased long-term incidence of abdominal cancers arising within the radiation field. They claimed that further work is required to ascertain whether there is a link of radiation exposure of patients during EVAR and endograft surveillance with differential late cancer risk. The aim of the present study was to investigate whether such an increased risk of EVAR compared with OAR for development of later abdominal and overall cancer was also seen in a large cohort of patients registered with a major health insurance company in Germany. This study addresses long-term patient survival after elective endovascular (EVAR) or open repair (OAR) of an intact abdominal aortic aneurysm (AAA) and relates it to postoperative cancer incidence.

## Patients and methods

All patients insured with AOK Die Gesundheitskasse (largest statutory health insurance company in Germany with a market share of 37%) undergoing elective surgery for an intact AAA either with EVAR (*n* = 14,218; 75.6%) or OAR (*n* = 4584; 24.4%) between 1/1/2010 and 31/12/2016 were included in this study. Patients with cancer history (*n* = 1881) were excluded from the study. All available data from cancer-free patients was included in this study. Cancer was recognized if a cancer-specific ICD was found in the insurance database. The end date of follow-up for all patients was 31/12/2018. Patients who underwent EVAR or OAR in 2010 were thus followed for 9 years and those from 2016 for 2 years (minimum follow-up, 0 months; maximum follow-up, 107 months; mean, 77 months). EVAR patients were followed for a mean time of 76 months and OAR patients for a mean time of 79 months. The mean follow-up for cancer-free patients was 80 months and for patients with cancer 67 months. Because health insurance data was analyzed, there were no patients lost to follow-up.

ICD and OPS codes were used to convert data regarding diagnosis, comorbidity, risk factors, and therapeutic approach into a comprehensive database using SPSS 27 (IBM Deutschland GmbH, Ehningen, Germany). ICD coding was used to tabulate the different types of cancer. The ICD codes for solid and hematological tumors were investigated in the ICD-10-GM-2020 database. The codes used for overall cancer analysis were: C00-C26, C30-C34, C37-C41, C43, C45-C58, C60-C85, C88, and C90-C97. In addition, abdominal cancers were analyzed with C15-25, C54-56, C61-C62, C64-C67, and C74, as specified by Markar et al. [2].

### Statistics

To test the significance of nominal variables, the chi-square test was used. The *p* values correspond to the significance of Fisher’s exact test. *t* test was performed to test for significance in metric variables. In each case, a Levene test for variance equality was performed. Kaplan–Meier estimates were used to show survival probabilities and cancer incidence up to 9-year follow-up in each group. Comparisons were made by a log-rank analysis. A univariate Cox proportional model was performed prior to a multivariate Cox proportional model to evaluate an independent effect of patient comorbidities, age, and type of surgery on postoperative tumor incidence. All analyzed comorbidities were diagnosed prior to the initial aneurysm operation. Multivariate Cox proportional models were used to evaluate possible confounding factors for the survival.

The designated level of significance was *p* < 0.050.

## Results

### Patients

Sixteen thousand eighty six patients were male (85.6%) and 2716 female (14.4%) (*p* < 0.001) (Table 1). The mean age of males at surgery was 72.1 years (median 73, min. 33, max. 98, SD 8.5). Females were significantly older than males, with a mean of 75.5 years (median 76, min. 16, max. 95, SD 8.3) (*p* < 0.001). Patients receiving EVAR (*n* = 14,218) were 73.4 years old on average (median 74, min. 38, max. 98, SD 8.4), and patients receiving OAR (*n* = 4584) were 70.2 years old (median 71, min. 16, max. 96, SD 8.7) (*p* < 0.001). More of the analyzed comorbidities were diagnosed in EVAR patients. 53.1% of EVAR patients compared to 41.6% of OAR patients suffered from arterial hypertension (*p* < 0.001). More EVAR patients were also diagnosed with COPD (13.9% vs. 10.1% *p* ≤ 0.001) or renal insufficiency stage 3–5 (10.4% vs. 7.0% *p* ≤ 0.001).

**Table 1:** Differences in characteristics and comorbidities between patients treated with EVAR or OAR

Characteristics and comorbidities	EVAR (*n* = 14,218)	OAR (*n* = 4584)	*p* value
Male sex, *n* (%)	12,262 (86.2)	3824 (83.4)	< .001
Female sex, *n* (%)	1956 (13.8)	760 (16.6)	< .001
Age, mean ± SD in years, median (min–max)	73.4 ± 8.4, 74 (38–98)	70.2 ± 8.7, 71 (16–96)	< .001
Age men, mean ± SD in years, median (min–max)	72.9 ± 8.4, 74 (38–98)	69.7 ± 8.5, 71 (33–96)	< .001
Age women, mean ± SD in years, median (min–max)	76.5 ± 7.9, 77 (40–95)	72.9 ± 8.8, 74 (16–90)	< .001
History of myocardial infraction, *n* (%)	1897 (13.3)	408 (8.9)	< .001
History of stroke, *n* (%)	674 (4.7)	175 (3.8)	.009
History of intracerebral bleeding, *n* (%)	51 (0.4)	10 (0.2)	.182
History of TIA, *n* (%)	355 (2.5)	89 (1.9)	.035
Arterial hypertension, *n* (%)	7543 (53.1)	1906 (41.6)	< .001
Dyslipoproteinemia, *n* (%)	4675 (32.9)	1172 (25.6)	< .001
Diabetes mellitus type 2, *n* (%)	2443 (17.2)	509 (11.1)	< .001
COPD, *n* (%)	1978 (13.9)	463 (10.1)	< .001
Renal insufficiency (stages 3–5), *n* (%)	1481 (10.4)	320 (7.0)	< .001
Left heart insufficiency, *n* (%)	1832 (12.9)	401 (8.7)	< .001
PAD (Fontaine stages 3–4), *n* (%)	599 (4.2)	177 (3.9)	.306

*EVAR*, endovascular aneurysm repair; *OAR*, open aneurysm repair; *SD*, standard deviation; *TIA*, transient ischemic attack; *COPD*, chronic obstructive pulmonary disease; *NYHA*, New York Heart Association *Renal insufficiency stage 3–5*; glomerular filtration rate under 60 ml/min/1.73 m^2^; *PAD*, peripheral artery disease

### Cancer incidence

**Fig. 1:**
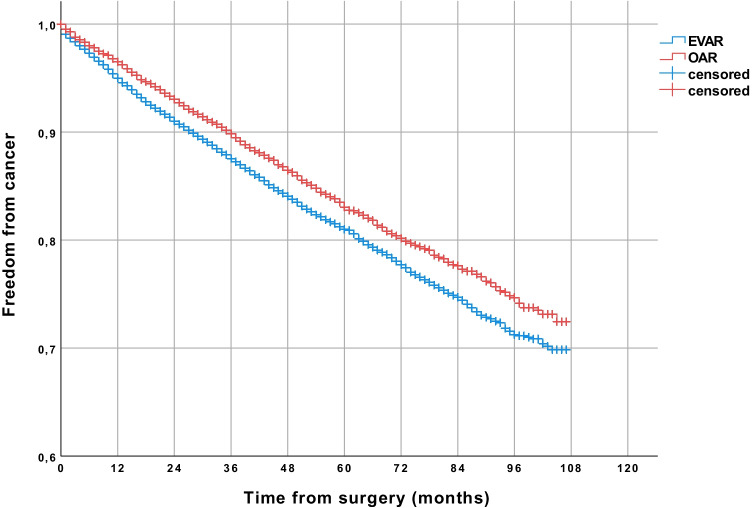
Comparison of patients receiving endovascular aneurysm repair (EVAR) or open abdominal aortic aneurysm repair (OAR), showing a higher rate of freedom from overall cancer in the long-term follow-up in the OAR group (Kaplan–Meier estimated)

**Fig. 2:**
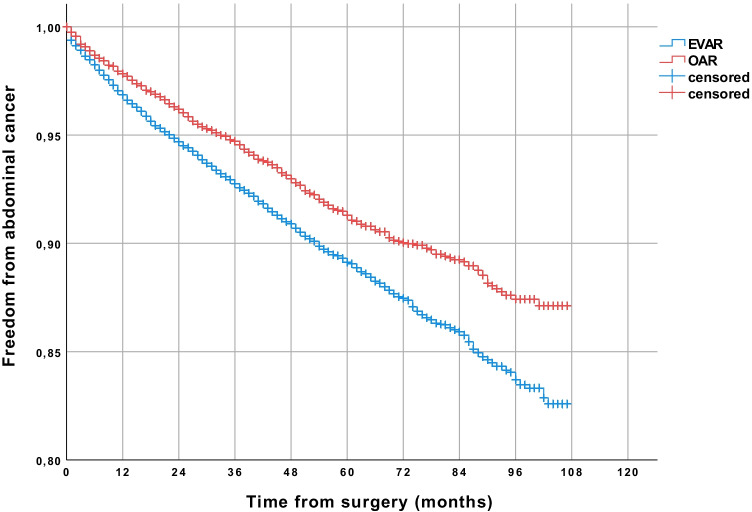
Comparison of patients receiving endovascular aneurysm repair (EVAR) or open abdominal aortic aneurysm repair (OAR), showing a higher rate of freedom from abdominal cancer in the long-term follow-up in the OAR group (Kaplan–Meier estimated)

In unadjusted data, 29.5% of all patients developed cancer after a follow-up period of up to 9 years (Kaplan–Meier estimated). More patients developed cancer after EVAR (30.1%) than after OAR (27.6%) (*p* < 0.001) (Table 2). After 9 years of follow-up, freedom from overall cancer was 72.4% in the OAR group and 69.9% in the EVAR group (*p* < 0.001) (Fig. 1), and freedom from abdominal cancer was 87.1% and 82,6%, respectively (Fig. 2) (*p* < 0.001). Postoperative cancer incidence was higher in men (30.8%) than in women (20.9%) (*p* < 0.001). 31.6% of men developed cancer after EVAR and 28.6% after OAR (*p* < 0.001). A significant difference regarding cancer incidence was not seen in women (EVAR 20.1%, OAR 22.1%) (*p* = 0.991). In the Cox regression analysis, EVAR was associated with a higher risk of abdominal cancer (HR 1.20; 95% CI 1.07–1.35, *p* = 0.002). Male gender (HR 2.24; 95% CI 1.86–2.68, *p* < 0.001) and age over 70 years (HR 1.62; 95% CI 1.46–1.80, *p* < 0.001) were also related to a higher risk of abdominal cancer. The analyzed comorbidities (cerebral infarction before surgery, arterial hypertension, diabetes, renal insufficiency) didn’t significantly affect the risk of abdominal cancer (Table 3).

**Table 2:** Cancer incidence after EVAR and OAR (all patients were cancer-free at time of repair)

Patients treated from 2010 to 2016	EVAR, *n* (%) (*n* = 14,218)	OAR, *n* (%) (*n* = 4584)	*p* value
Cancer incidence	30.1%	27.6%	*p* < .001
Cancer incidence patients < 70 years	24.9%	25.5%	*p* = .047
Cancer incidence patients > 70 years	33.3%	29.1%	*p* = .076
Cancer incidence men	31.6%	28.6%	*p* < .001
Cancer incidence women	20.1%	22.1%	*p* = .991
Abdominal cancer incidence	17.4%	12.9%	*p* < .001
Abdominal cancer incidence patients < 70 years	13.1%	11.2%	*p* = .016
Abdominal cancer incidence patients > 70 years	20.1%	14.3%	*p* = .003
Abdominal cancer incidence men	18.8%	13.7%	*p* < .001
Abdominal cancer incidence women	8.2%	8.7%	*p* = .876

*EVAR*, endovascular aneurysm repair; *OAR*, open aneurysm repair

**Table 3:** Hazard ratio (HR) and proportional hazard model (multiple variate analysis) for abdominal cancer incidence

Covariate	HR	95% CI	*p* value
Male (vs. female)	2.24	1.86–2.68	*p* < .001
Age over 70 (vs. under 70)	1.62	1.46–1.80	*p* < .001
EVAR (vs. OAR)	1.20	1.07–1.35	*p* = .002
Stroke	1.21	0.96–1.51	*p* = .101
Arterial hypertension	1.10	0.99–1.22	*p* = .065
Diabetes mellitus type 2	1.06	0.92–1.22	*p* = .417
Renal insufficiency stages 3–4	1.10	0.92–1.30	*p* = .298

*EVAR*, endovascular aneurysm repair; *OAR*, open aneurysm repair; *HR*, hazard ratio; CI, confidence interval; *Renal insufficiency stages 3–5*, glomerular filtration rate under 60 ml/min/1.73 m^2^

For overall cancer incidence, male gender (HR 1.56; 95% CI 1.39–1.75, *p* < 0.001) and age over 70 years (HR 1.49; 95% CI 1.38–1.61, *p* < 0.001) were significant risk factors. COPD was also linked to a significantly higher cancer risk (HR 1.25; 95% CI 1.12–1.39, *p* < 0.001). In contrast, EVAR was only by trend associated with a higher risk of overall cancer incidence (HR 1.09; 95% CI 1.00–1.18, *p* = 0.051) (Table 4).

**Table 4:** Hazard ratio (HR) and proportional hazard model (multiple variate analysis) for overall cancer incidence

Covariate	HR	95% CI	*p* value
Male (vs. female)	1.56	1.39–1.75	*p* < .001
Age over 70 (vs. under 70)	1.49	1.38–1.61	*p* < .001
EVAR (vs. OAR)	1.09	1.00–1.18	*p* = .051
Stroke	1.17	0.99–1.39	*p* = .073
Arterial hypertension	1.03	0.95–1.11	*p* = .519
Diabetes mellitus type 2	1.01	0.91–1.12	*p* = .810
COPD	1.25	1.12–1.39	*p* < .001
Renal insufficiency stages 3–4	1.13	0.99–1.28	*p* = .061
Peripheral arterial vascular disease stages 3–4	1.24	1.04–1.48	*p* = .019

*EVAR*, endovascular aneurysm repair; *OAR*, open aneurysm repair; *COPD*, chronic obstructive pulmonary disease; *HR*, hazard ratio; *CI*, confidence interval

### Survival of patients with and without cancer (total cohort)

**Fig. 3:**
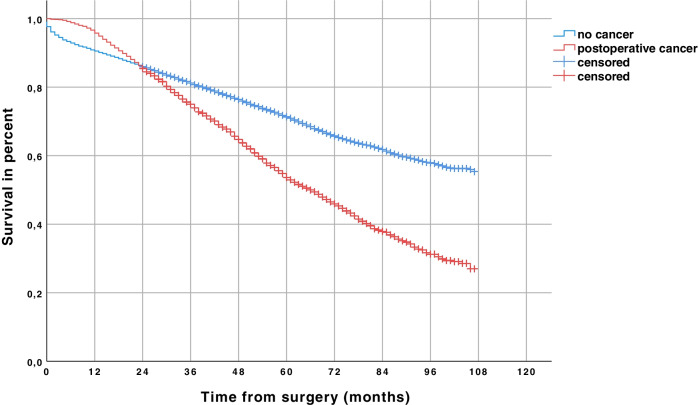
Survival of patients with cancer and cancer-free patients during long-term follow-up (Kaplan–Meier estimated)

Estimated 55.4% of cancer-free patients (56.9% of men and 47.4% of women; *p* < 0.001) survived compared to 27.0% of patients with cancer (26.9% of men and 26.9% of women; *p* = 0.796) (*p* < 0.001) (Fig. 3).

### Survival EVAR vs. OAR

**Fig. 4:**
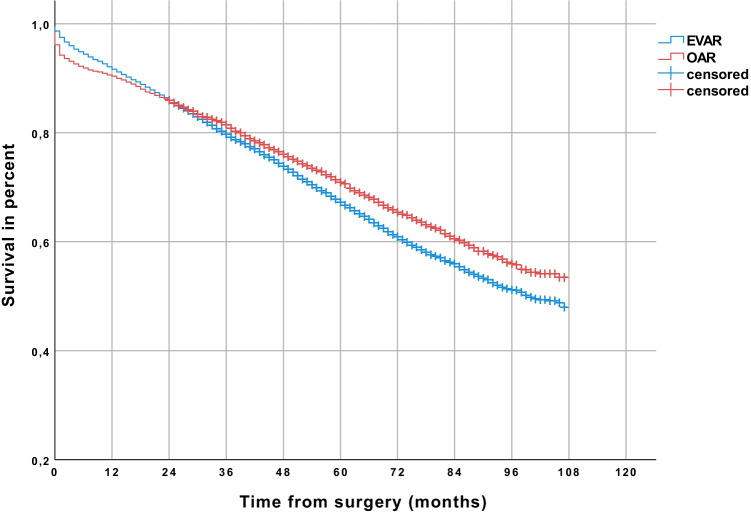
Comparison of patients receiving endovascular aneurysm repair (EVAR) or open abdominal aortic aneurysm repair (OAR), showing a higher survival rate in the long-term follow-up in the OAR group (Kaplan–Meier estimated)

**Fig. 5:**
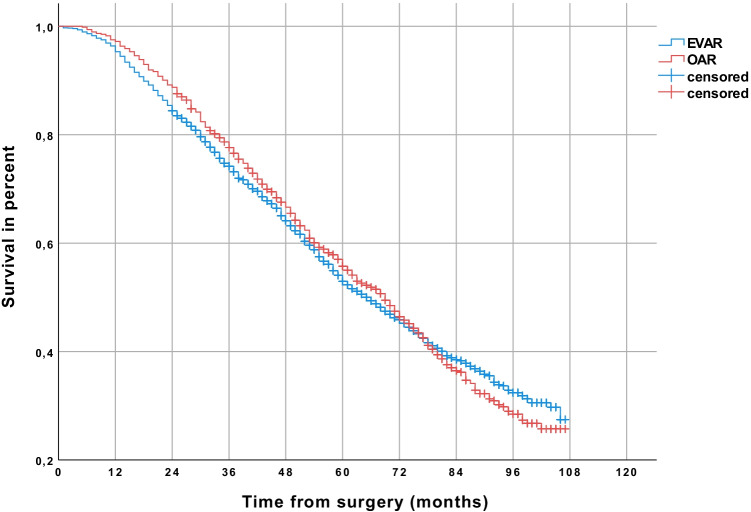
Patients with cancer in the follow-up: comparison of patients receiving endovascular aneurysm repair (EVAR) or open abdominal aortic aneurysm repair (OAR), showing no difference in the long-term survival of patients with cancer (Kaplan–Meier estimated)

**Fig. 6:**
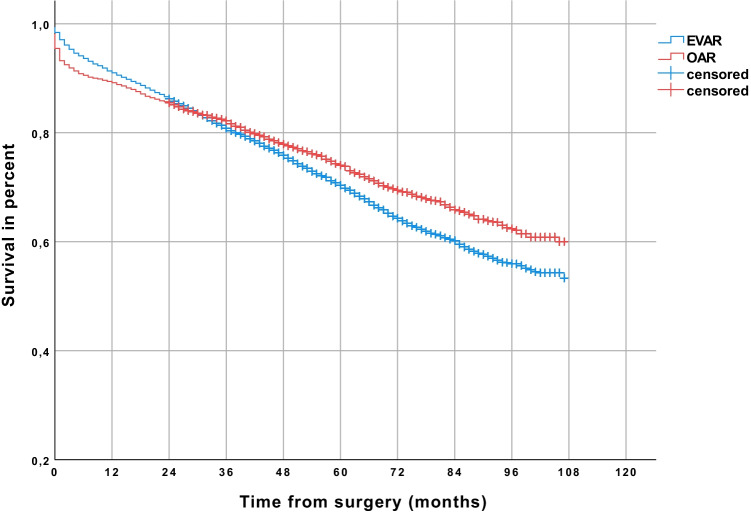
Cancer-free patients in the follow-up: comparison of patients receiving endovascular aneurysm repair (EVAR) or open abdominal aortic aneurysm repair (OAR), showing in the long-term follow-up higher survival of cancer-free patients in the OAR group (Kaplan–Meier estimated)

Regardless of cancer incidence, the estimated survival was 53.4% for patients who underwent OAR compared to 48.0% of patients who underwent EVAR (*p* < 0.001) (Fig. 4). In cancer patients (Fig. 5), the estimated survival did not differ significantly between EVAR (survival rate 27.5%) and OAR (survival rate 25.7%) (Fig. 5) (*p* = 0.933). In contrast, in cancer-free patients, the estimated survival was 60.0% after OAR as compared to 53.3% after EVAR (*p* < 0.001) (Fig. 6).

### Cox regression for survival

To adjust confounding factors like comorbidities, patient age, and treatment for survival, a multivariate Cox regression model was used, and hazard ratios (HR) with 95% confidence interval were calculated (Table 5). Major impact factors on survival were patient age (HR 2.29; 95% CI 2.15–2.43, *p* < 0.001) and cancer incidence (HR 1.68; 95% CI 1.59–1.78, *p* < 0.001). Comorbidities like arterial hypertension (HR 1.15; 95% CI 1.08–1.22, *p* < 0.001), dyslipoproteinemia (HR 0.82; 95% CI 0.77–0.87, *p* < 0.001), diabetes (HR 1.15; 95% CI 1.07–1.23, *p* < 0.001), COPD (HR 1.66; 95% CI 1.55–1.77, *p* < 0.001), renal insufficiency (HR 1.60; 95% CI 1.49–1.73, *p* < 0.001), left heart failure (HR 1.27; 95% CI 1.17–1.37, *p* < 0.001), and peripheral arterial vascular disease (HR 1.65; 95% CI 1.49–1.84, *p* < 0.001) had also a significant impact on survival. In contrast, the type of surgery and gender did not significantly influence overall survival.

**Table 5:** Hazard ratio (HR) and proportional hazard model (multiple variate analysis) for overall survival

Covariate	HR	95% CI	*p* value
Patients with cancer (vs. patients without cancer)	1.68	1.59–1.78	*p* < .001
Male (vs. female)	0.94	0.88–1.00	*p* = .061
Age over 70 (vs. under 70)	2.29	2.15–2.43	*p* < .001
EVAR (vs. OAR)	0.96	0.91–1.02	*p* = .219
Myocardial infarction	1.02	0.94–1.10	*p* = .716
Stroke	1.27	1.13–1.43	*p* = < .001
Intracerebral bleeding before operation	1.33	0.91–1.96	*p* = .145
TIA before operation	1.03	0.88–1.22	*p* = .713
Arterial hypertension	1.15	1.08–1.22	*p* < .001
Dyslipoproteinemia	0.82	0.77–0.87	*p* < .001
Diabetes mellitus type 2	1.15	1.07–1.23	*p* < .001
COPD	1.66	1.55–1.77	*p* < .001
Renal insufficiency stages 3–4	1.60	1.49–1.73	*p* < .001
Left heart failure	1.27	1.17–1.37	*p* < .001
Peripheral arterial vascular disease stages 3–4	1.65	1.49–1.84	*p* < .001

*EVAR*, endovascular aneurysm repair; *OAR*, open aneurysm repair; *TIA*, transient ischemic attack; *COPD*, chronic obstructive pulmonary disease; *HR*, hazard ratio; *CI*, confidence interval

## Discussion

In this cohort study (AOK insurance patients), including only patients with no history of cancer, 29.5% of patients who underwent EVAR or OAR developed cancer during a follow-up period of up to 9 years. An increased risk of abdominal cancer after EVAR compared with open AAA repair (HR 1.20; 95% CI 1.07–1.35, *p* = 0.002) but not of all cancers (HR 1.09; 95% CI 1.00–1.18, *p* = 0.051) was found. Cancer significantly worsened the long-term outcome after EVAR and OAR. In the adjusted data, the operation method had no significant impact on overall survival (HR 0.96; 95% CI 0.91–1.02, *p* = 0.219).

Late-onset cancer reported by Patel et al. [1] is lower than in the patient population presented here, but they reported cancer as cause of death rather than cancer incidence. Markar et al. [2] also reported cancer as cause of death and included patients with emergency intervention in their survey, which worsened the overall outcome. In their survey of the English Hospital Episode Statistics (HES) database from 2005 to 2013, they saw an increased risk of death due to carcinoma at follow-up up to 7 years (median 2.45 years) after EVAR compared with OAR (hazard ratio 1.09). Specifically, for abdominal tumors, the risk was higher after EVAR than after OAR (hazard ratio 1.14). However, when only electively treated patients were considered in their study, the risk was not higher after EVAR than after OAR. Cancer was the cause of death in 19.7% of all deaths after EVAR and in 20.9% of all deaths after OAR.

After more than 8 years of follow-up in the EVAR trial 1, mortality was lower after OAR than after EVAR [1]. This difference was not apparent in the OVER trial, where Lederle et al. [3] saw no significant differences in long-term survival after EVAR or OAR. In this trial, cancer was the cause of death in 18% after EVAR and in 19.5% after OAR. In a review of data from the English Hospital Episode Statistics from 2006 to 2015 (37,138 elective AAA patients; 15,523 OAR; 21,615 EVAR), Johal et al. [4] reported a 10-year mortality of 58.6% for the total cohort, with substantial subgroup differences depending on patient age and risk factors. Patients with EVAR had a higher long-term mortality risk, but Johal et al. did not examine the specific risk associated with cancer. In the investigation presented here, the patients age also significantly influenced their survival (age < 70 years vs. > 70 years; HR 2.29; 95% CI 2.15–2.43; *p* < 0.001). The best survival (Kaplan–Meier estimated) was seen in cancer-free patients < 70 years (76.2%) as compared to 43.5% in cancer-free patients > 70 years (*p* < 0.001).

Markar et al. [2] suggested that an increased risk of abdominal cancer was associated with EVAR rather than with open repair, whereas no such association was found for lung cancer and nonabdominal-related cancers. They speculated that radiation exposure during fluoroscopy may be associated with an increased long-term incidence of abdominal cancers arising within the radiation field. In addition, the widespread use of CT for abdominal investigation could be associated with an increased lifetime risk of cancer.

The extent of radiation exposure could not be measured in the study presented here, especially since this would also include data on outpatient diagnostics in the follow-up. Nevertheless, the data from this study confirms the observation of Markar et al. The incidence of abdominal cancers was significantly higher in patients who underwent EVAR as compared to patients who underwent OAR (HR 1.20; 95% CI 1.07–1.35; *p* = 0.002). The reasons for this must remain open; causation cannot be inferred from the available nonrandomized and observational data. We agree with Markar et al. that the differential cancer risk should be further explored in alternative national populations.

In our study, cancer incidence played a decisive role on survival after elective AAA repair. Patients without cancer had an estimated survival rate of 55.4%, compared to only 27.0% in patients with cancer (*p* < 0.001) (Fig. 3). In the multivariate analysis, EVAR was no risk factor for survival (HR 0.96; 95% CI 0.91–1.02; *p* = 0.219). Survival was mainly influenced by patient age and comorbidities but not by the type of surgery (Table 5).

It was expected that patients with cancer would have a worse prognosis than those without malignancy, but the significant difference between patients with and without cancer has not been underscored in reports to date. Cancer incidence, without adequate analysis in the different collectives, may have contributed to conflicting conclusions about long-term survival after EVAR or OAR in meta-analyses. For example, in a meta-analysis of 7 randomized trials, Antoniou et al. [5] found significantly lower hospital mortality with EVAR compared to OAR, but long-term aneurysm-related mortality, reintervention rates, and rupture rates were higher after EVAR than after OAR. Li et al. [6] reached a similar conclusion based on 54 studies and 203,246 patients. Bulder et al. [7] confirmed a lower in-hospital mortality after EVAR in another meta-analysis but, in the long-term over 10 years, found no significant differences in survival between EVAR and OAR.

In the present study, mortality and cancer incidence were followed for up to 9 years in a non-selected patient population having received EVAR or OAR. The strength of this study is the complete database for all patients treated in the reviewed period, so that survival at the end date of follow-up could be exactly established.

### *Limitations*

One limitation of this study is that the available data include a limited portion of the overall care of patients with AAA in Germany and may reflect the market potential of the health insurer and the social structure of its clientele. Whether patients of private health insurance companies have the same risk factors in identical frequency has not been investigated. However, the AOK insures more than 26 million persons and is the largest statutory health insurer in Germany with a market share of 37%. The cause of death was not reported; aneurysm-related and cancer-related deaths therefore cannot be differentiated. As we analyzed health insurance data, coding errors may be possible. We also must note that in the analysis of the unadjusted data, the estimated survival after OAR as compared to EVAR was significantly better (estimated 53.4% vs. 48.0%; *p* < 0.001) (Fig. 4). This was not confirmed in the multivariate analysis. No information on follow-up appointments and medical examinations were analyzed in this study. This leads to no information on CT exposure in the follow-up period. An explanation for the increased risk of abdominal cancer with EVAR (for example, radiation exposure) cannot be given.

## Conclusion

This study suggests in the long-term an increased risk of abdominal cancer after EVAR compared with open AAA repair (HR 1.20; 95% CI 1.07–1.35, *p* = 0.002) but not of all cancers (HR 1.09; 95% CI 1.00–1.18, *p* = 0.051). Cancer significantly worsened the long-term outcome after EVAR and OAR. In the unadjusted data, the estimated overall survival rate was 60.0% with OAR and 53.3% with EVAR (*p* < 0.001). However, in the multiple variate analysis, no significant differences in overall survival between the two interventions were found (HR 0.96; 95% CI 0.91–1.02, *p* = 0.219). The higher abdominal cancer incidence with EVAR can affect quality of life including oncological therapy and therefore should be considered when determining the indication for surgery, and the patient should be informed about it.
